# Current Advances in γδ T Cell-Based Tumor Immunotherapy

**DOI:** 10.3389/fimmu.2017.01401

**Published:** 2017-10-27

**Authors:** Elena Lo Presti, Gabriele Pizzolato, Eliana Gulotta, Gianfranco Cocorullo, Gaspare Gulotta, Francesco Dieli, Serena Meraviglia

**Affiliations:** ^1^Dipartimento di Biopatologia e Metodologie Biomediche, University of Palermo, Palermo, Italy; ^2^Central Laboratory of Advanced Diagnosis and Biomedical Research (CLADIBIOR), University of Palermo, Palermo, Italy; ^3^Humanitas University, Rozzano-Milano, Italy; ^4^Dipartimento di Discipline Chirurgiche ed Oncologiche, University of Palermo, Palermo, Italy

**Keywords:** γδ T cells, immunotherapy, adoptive transfer, Zoledronate, immunoevasion

## Abstract

γδ T cells are a minor population (~5%) of CD3 T cells in the peripheral blood, but abound in other anatomic sites such as the intestine or the skin. There are two major subsets of γδ T cells: those that express Vδ1 gene, paired with different Vγ elements, abound in the intestine and the skin, and recognize the major histocompatibility complex (MHC) class I-related molecules such as MHC class I-related molecule A, MHC class I-related molecule B, and UL16-binding protein expressed on many stressed and tumor cells. Conversely, γδ T cells expressing the Vδ2 gene paired with the Vγ9 chain are the predominant (50–90%) γδ T cell population in the peripheral blood and recognize phosphoantigens (PAgs) derived from the mevalonate pathway of mammalian cells, which is highly active upon infection or tumor transformation. Aminobisphosphonates (n-BPs), which inhibit farnesyl pyrophosphate synthase, a downstream enzyme of the mevalonate pathway, cause accumulation of upstream PAgs and therefore promote γδ T cell activation. γδ T cells have distinctive features that justify their utilization in antitumor immunotherapy: they do not require MHC restriction and are less dependent that αβ T cells on co-stimulatory signals, produce cytokines with known antitumor effects as interferon-γ and tumor necrosis factor-α and display cytotoxic and antitumor activities *in vitro* and in mouse models *in vivo*. Thus, there is interest in the potential application of γδ T cells in tumor immunotherapy, and several small-sized clinical trials have been conducted of γδ T cell-based immunotherapy in different types of cancer after the application of PAgs or n-BPs plus interleukin-2 *in vivo* or after adoptive transfer of *ex vivo*-expanded γδ T cells, particularly the Vγ9Vδ2 subset. Results from clinical trials testing the efficacy of any of these two strategies have shown that γδ T cell-based therapy is safe, but long-term clinical results to date are inconsistent. In this review, we will discuss the major achievements and pitfalls of the γδ T cell-based immunotherapy of cancer.

## Introduction

T cells carrying the γδ T cell receptor (TCR) are a minor lymphocyte population that accounts for 2–5% of CD3 T cells in the peripheral blood, but predominate in several anatomic sites such as the intestine and the skin. There are two major γδ T cell subsets in humans which are distinguished based on the δ chain they use to make their TCR: T cells expressing the Vδ2 gene paired with the Vγ chain (Vγ9) are the great majority of the γδ T cell population in the peripheral blood and secondary lymphoid organs of healthy individuals. In contrast, γδ T cells expressing the Vδ1 gene, paired off with different Vγ elements, are the predominant γδ T cell subset in epithelia (skin and mucosa). Finally, a third subset of γδ T cells expressing the Vδ3 chain abound in the liver (1).

Vδ1 T cells have a largely private TCR repertoire with different clonotypes present in each individual, while the Vγ9Vδ2 repertoire has limited complexity with invariant Vγ9-JP sequences common to multiple individuals, and many CDR3δ2 sequences although are relatively private compared with TCRγ9 lengths, are shared between individuals (2, 3). Therefore, the Vγ9Vδ2 T cell population expresses a TCR with very limited variability, suggesting recognition of a limited set of antigens.

Antigen recognition by γδ T cells is a field of intense research. Vγ9Vδ2 T cells recognize non-peptidic phosphorylated intermediates of the non mevalonate pathway of isoprenoid biosynthesis called phosphoantigens (PAgs), in the absence of processing, presentation, and major histocompatibility complex (MHC) restriction (4). PAgs are synthesized in mammalian cells through the mevalonate pathway (5), but PAg concentrations required for Vγ9Vδ2 T cell activation are not achieved in physiological conditions, but only after infections or tumor transformation (6). Therefore, from this point of view, the Vγ9Vδ2 TCR works in a similar way to a pattern-recognition receptor, which senses metabolic changes found in transformed or infected cells.

Intracellular PAg levels can be modulated by drugs. Thus, aminobisphosphonates (n-BPs) such as Zoledronate, widely used in the clinic for the treatment of osteoporosis and bone metastasis, inhibit farnesyl pyrophosphate synthase (FPPS), a downstream enzyme of the mevalonate pathway, thereby causing accumulation of upstream PAgs and thus favoring Vγ9Vδ2 T cell activation (7, 8). Conversely, statins inhibit hydroxy-methylglutaryl-CoA reductase (HMGCR), the upstream enzyme of the mevalonate pathway, and significantly reduce PAgs production and Vγ9Vδ2 T cell activation (9).

Vδ1 T cells recognize MHC class I-related molecule A (MICA), MHC class I-related molecule B (MICB), and UL16-binding proteins (ULBPs) molecules, a group of proteins expressed on stressed and tumor cells (10, 11), and the MHC-related class Ib molecules CD1c and CD1d, which are typically involved in glycolipid presentation (12, 13). However, as Vδ1 T cells constitutively express natural-killer group 2, member D (NKG2D), the “true” receptor of MICA and MICB, it is still to be determined if Vδ1 T cell recognition of MICA and MICB is mediated by the TCR or by NKG2D. Moreover, Vδ1 T cells can also be activated by engagement of natural cytotoxicity receptors (NCRs, such as NKp30 and NKp44) by yet unidentified ligands (14). Similar to Vδ1 T cells, Vδ3 T cell ligands are poorly unknown and there is only one study showing that these cells are activated by CD1d possibly bound to a yet unidentified glycolipid (15).

Phosphoantigens recognition by Vγ9Vδ2 T cells requires butyrophilin (BTN) 3A1 (also called CD277) (16), but how PAgs interact with BTN3A1 and how the PAg/BTN3A1 complex in turn interacts with the Vγ9Vδ2 TCR is a matter of debate. Initial studies by Vavassori et al. (17) found a PAg-binding site located in the extracellular domain of BTN3A1, but a subsequent study by Adams and coworkers (18) found that PAgs bind to the intracellular domain of BTN3A1, leading to the possibility that intracellular PAgs provoke a conformational change of BTN3A1, which allows its extracellular domains to interact with the reactive Vγ9Vδ2 TCR.

Vγ9Vδ2 T cells express several cell surface molecules correlated with distinct functional differentiation phenotypes. The combined use of CD27 and CD45RA permits identification of “naive” and “central memory” subsets of Vγ9Vδ2 T cells (T_Naive_, CD45RA^+^CD27^+^; T_CM_, CD45RA^−^CD27^+^) that circulate between the blood and secondary lymphoid organs, but are excluded from peripheral tissues and lack effector function; and “effector memory” (T_EM_, CD45RA^−^CD27^−^) and “terminally differentiated” (T_EMRA_, CD45RA^+^CD27^−^) subsets that circulate between the blood and peripheral tissues, are recruited to sites of inflammation and immediately perform effector function (19).

While T_Naive_ and T_CM_ cells readily respond to PAg stimulation, T_EM_ and T_EMRA_ respond to homeostatic cytokines as interleukin (IL)-15 (20) and may acquire highly diverse effector functions in the presence of polarizing cytokines (21). In general, circulating Vγ9Vδ2 T cells have a Th1 pattern of cytokine production (21), but under certain conditions they polarize to Th2 (22, 23), Th17 (24–26), follicular T helper (27, 28), Th9 (29), and T regulatory (Treg) cells (30). Such a flexibility emphasizes the capacity of Vγ9Vδ2 T cells to efficiently participate to immune responses to different antigen challenges.

## Rationale for Harnessing γδT Cells in Cancer Immunotherapy

In the following section, we will briefly summarize the rationale for harnessing γδ T cells in cancer immunotherapies.

**Figure 1:**
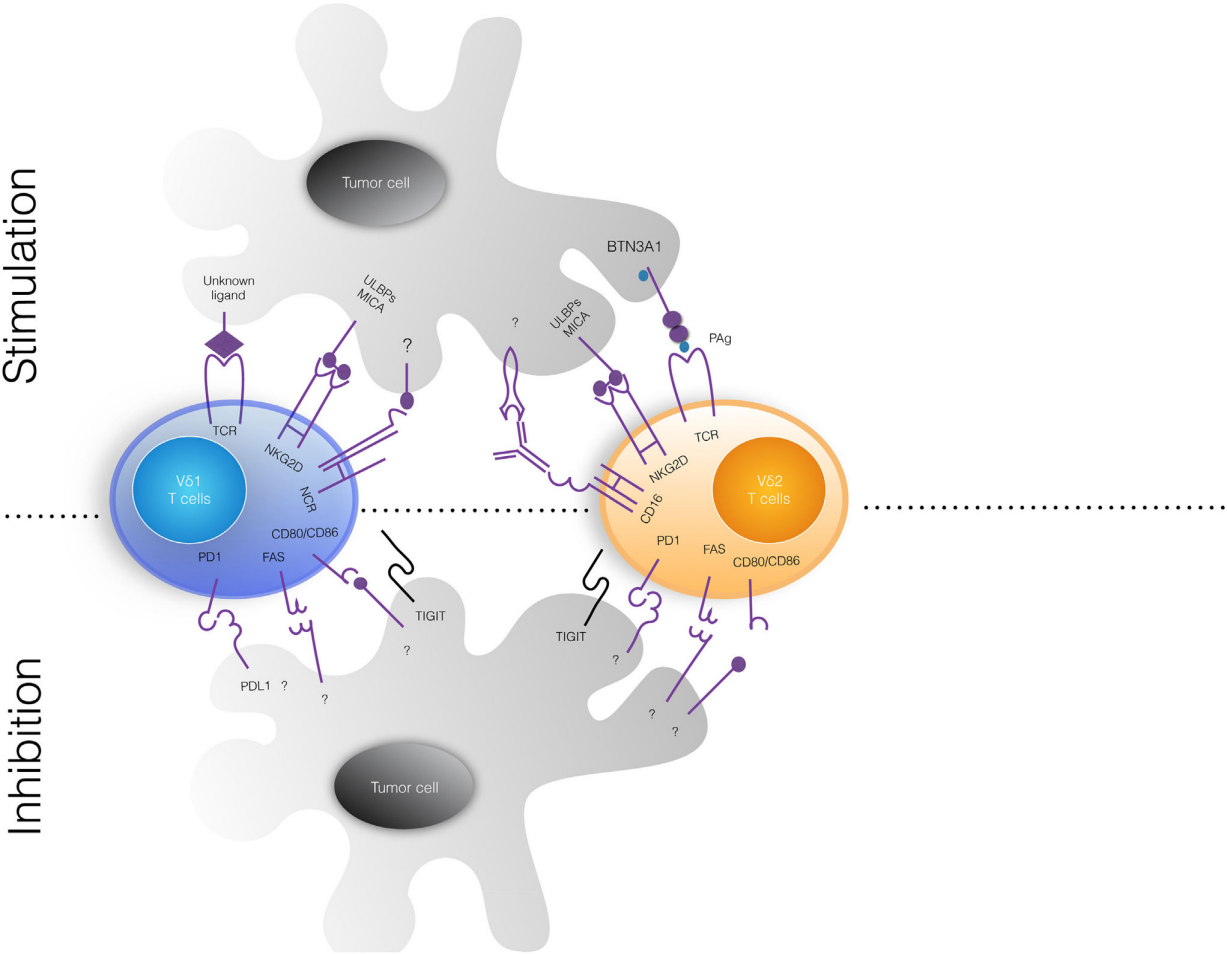
Tumor cell ligands recognized by human γδ T cells. The upper and lower panels show stimulatory and inhibitor signals delivered by tumor cells to Vδ1 (left) and Vδ2 (right) γδ T cell subsets. Vγ9Vδ2 T cells recognize *via* their TCR non-peptidic phosphoantigens (PAgs) and BTN3A1, while Vδ1 T cell receptor (TCR) ligands are not defined yet. Both γδ T cell subsets constitutively express surface natural cytotoxicity cell receptors (NCRs) that bind MICA/MICB and ULBPs, frequently expressed on tumor cells. Upon activation, Vγ9vδ2 T cells express fragment crystallizable receptor for IgG (FcγRIII; also known as CD16) that can bind therapeutic antibodies and mediate antibody-dependent cell-mediated cytotoxicity phenomena. Inhibitor signals delivered by tumor cells have not been well characterized. MICA/B, MHC class I-related chain A/B; ULBP, UL16-binding protein; BTN3A1, butyrophilin 3A1.

(1) The major objective of immunotherapy is the generation of a long-lasting efficient antitumor response, particularly mediated CD8 cytotoxic T cells, but also by CD4 T cells (31, 32). Nonetheless, despite efforts, durable responses are only rarely achieved and moreover tumors often develop strategies to escape immune responses (33). In contrast to CD4 or CD8 T cells, γδ T cells have unique features which make them good candidates for effective tumor immunotherapy: they do not require MHC restriction and co-stimulation and they recognize antigens shared by a variety of stressed and tumor cells, making it possible for a single γδ T cell to target a vast array of tumor cells. Hence, recognition of commonly shared tumor antigens in the absence of MHC restriction provides the rationale for application of γδ T cell-based therapy to a wide range of tumors and in patients with different MHC molecules (34).
(2) A distinctive feature of T lymphocytes equipped with antitumor potential is their ability to secrete appropriate cytokines. Typically, activated γδ T cells secrete interferon (IFN)-γ and tumor necrosis factor (TNF)-α, two cytokines with cytotoxic and antitumor activities (35–37).
(3) A large body of studies have demonstrated that γδ T cells kill *in vitro* a broad array of tumor cells, while sparing normal cells (34), and display antitumor activity in mouse models *in vivo* (34). The cytotoxic activity of γδ T cells against tumor cells is strictly dependent on augmented production of PAgs (38), which partly relies on increased expression of HMGCR (38). Moreover, intracellular PAgs levels can be substantially increased by n-BPs (13–15, 38), thereby promoting activation of Vγ9Vδ2 T cells (38). Killing may also be reinforced by the tumor cell expression of NCRs (39) and/or NKG2D ligands (such as MICA, MICB, and ULBPs) (40–42) or by antibody-dependent cell-mediated cytotoxicity (ADCC) mediated by CD16 interacting with antibody-coated tumor cells (43) (Figure 1).
  Whatever the mechanism of γδ T cell recognition of tumor target cells, killing involves the perforin/granzyme (44) and TNF-related apoptosis-inducing ligand (TRAIL) (45) pathways, and Fas/FasL interaction (46). The choice of the mechanism is mostly dictated by the nature of the target cell itself (47). For instance, we previously found that colon cancer stem cells (CSCs), which are typically resistant to γδ T cell-mediated cytotoxicity, are efficiently killed upon sensitization with Zoledronate (48). Killing of Zoledronate-treated colon CSCs was abrogated by anti-CD3 or anti-γδ TCR monoclonal antibodies (mAbs), or mevastatin, which inhibits HMGCR and prevents PAg accumulation, and by Concanamycin A that blocks degranulation, indicating that Vγ9Vδ2 T cells recognize Zoledronate-treated colon CSCs by the TCR interacting with PAgs and utilize the perforin pathway to kill them (48). The colon CSCs are usually resistant also to chemotherapy, but we unexpectedly found that pretreatment with 5-Fluorouracil and Doxorubicin sensitizes colon CSCs to killing by Vγ9Vδ2 T cells. However, killing of chemotherapy-sensitized colon CSCs by Vγ9Vδ2 T cells was inhibited by anti-NKG2D mAb and by blocking TRAIL interaction with its death receptor 5 (DR5), indicating that Vγ9Vδ2 T cells recognize chemotherapy-treated colon CSCs by NKG2D interaction with MICA/B or ULBPs and kill them through mechanisms involving TRAIL interaction with DR5 (49).
(4) In order for T lymphocytes to interact with tumor cells they should be capable to infiltrate tumors. Tumor-infiltrating leukocytes are found in a several different solid tumors (50) and include both myeloid (granulocytes, macrophages, and myeloid-derived suppressor cells) and lymphoid (T, B, and NK) cells, each of which impacts differently on tumor prognosis (51). Tumor-infiltrating Vγ9Vδ2 T lymphocytes have been detected in several types of cancer (52), but their clinical relevance has remained long obscure because of inconsistent results. However, analysis of expression signatures from ~18,000 human tumors with overall survival outcomes across 39 malignancies identified tumor-infiltrating γδ T cells as the most significant favorable cancer-wide prognostic signature (53). Similarly, our own results of data mining transcriptomes and clinical files from a large cohort of colorectal cancer samples (*n* = 585), revealed that the 5-year disease-free survival probability was significantly higher in patients with high number of tumor-infiltrating γδ T cells (54).
(5) Two synthetic drugs, the PAg bromohydrin pyrophosphate (BrHPP) and the n-BP Zoledronate, activate human Vγ9Vδ2 T lymphocytes *in vitro* and in clinical trials *in vivo*. BrHPP is produced as good manufacturing practice grade for use in humans under the name Phosphostim (55). Zoledronate, a third generation n-BP used to treat osteoporosis and bone metastasis, inhibits FPPS and causes accumulation of endogenous PAgs which thus reach the threshold required for Vγ9Vδ2 T cell activation (56). Second generation n-BPs, such as Pamidronate, Alendronate, and Risedronate, have similar activities of Zoledronate but at higher concentrations (55). Of note, *in vitro* and *in vivo* expansion of Vγ9Vδ2 T cells by either PAgs or n-BPs requires exogenous IL-2.

Overall, the above functional aspects of γδ T cell biology, have led to their utilization in cancer immunotherapy, and two strategies have been developed: (1) *in vivo* administration of PAgs or n-BPs that activate Vγ9Vδ2 T cells and (2) adoptive transfer of *ex vivo*-expanded Vγ9Vδ2 T cells. Several small-sized phase I clinical trials have assessed the safety and efficacy of these two strategies in patients with various tumor types, and available data suggest that Vγ9Vδ2 T cell-based immunotherapy is well tolerated and may give some clinical benefit to patients, thus providing a proof of principle for its utilization in addition to conventional therapies (57).

In the following sections, we will review the major achievements and pitfalls of the Vγ9Vδ2 T cell-based immunotherapy.

## Results from Clinical Trials Based on *In Vivo* Activation of γδ T Cells

A survey of clinical trials based on *in vivo* activation of γδ T cells in different types of cancer is shown in Table 1.

**Table 1:** Survey of clinical trials based on *in vivo* activation of γδ cells.

Author	Year	Tumor	Treatment	Reference
Wilhelm et al.	2003	MM, NHL	Pamidronate + IL-2	(58)
Dieli et al.	2003	Prostate, breast	Zoledronate	(8)
Dieli et al.	2007	Prostate	Zoledronate/Zoledronate + IL-2	(59)
Meraviglia et al.	2010	Breast	Zoledronate + IL-2	(60)
Bennouna et al.	2010	Solid tumors	BrHPP + IL-2	(61)
Gertner-Dardenne et al.	2010	FBCL	Rituximab + BrHPP + IL-2	(62)
Lang et al.	2011	RCC	Zoledronate + IL-2	(63)
Kunzmann et al.	2012	RCC, MM, AML	Zoledronate + IL-2	(64)
Pressey et al.	2016	Neuroblastoma	Zoledronate + IL-2	(65)

*MM, multiple myeloma; NHL, non-Hodgkin lymphoma; FBCL, follicular B-cell lymphoma; RCC, renal cell cancer; AML, acute myeloid leukemia; BrHPP, bromohydrin pyrophosphate; IL, interleukin*.

Since B-cell type non-Hodgkin lymphoma (NHL) and multiple myeloma (MM) are highly sensitive to lysis by Vγ9Vδ2 T cells *in vitro*, a pioneering study by Wilhelm and colleagues (58) analyzed *in vivo* the toxicity, Vγ9Vδ2 T cell activation and anti-lymphoma activity of Pamidronate and IL-2 in 19 patients with NHL or MM. Ten patients received Pamidronate followed by IL-2, but neither Vγ9Vδ2 T cell activation nor response to treatment were observed. Therefore, a second group of nine patients was selected for *in vitro* Vγ9Vδ2 T cell response to Pamidronate and IL-2 and was treated with Pamidronate followed by increasing doses of IL-2. Significant *in vivo* expansion of Vγ9Vδ2 T cells was detected in this group, and three patients achieved objective responses. This was the first study demonstrating activation of Vγ9Vδ2 T cells in patients with B-cell lymphomas by Pamidronate and low-dose IL-2 was well tolerated and induced a clinical response; moreover, the immunologic and clinical outcome could be nicely predicted by Vγ9Vδ2 T cell proliferation *in vitro*.

At the same time as the aforementioned study, we performed an observational study in nine cancer patients with bone metastases to determine if Zoledronate affected activation and maturation of circulating Vγ9Vδ2 T cells *in vivo* (8). The results of that study showed that Zoledronate-induced the *in vivo* differentiation of Vγ9Vδ2 T cells to the T_EM_ subset producing IFN-γ. Therefore, and based on this, we then conducted a phase I clinical trial in 18 patients with metastatic hormone-refractory prostate cancer (59). Patients were randomized into two groups, one receiving Zoledronate alone and the other receiving Zoledronate together with low-dose IL-2 subcutaneously (s.c.). The treatments were well tolerated and a significant clinical response was observed in the group receiving Zoledronate and IL-2 during the 1-year follow-up, which correlated with sustained elevated numbers of blood Vγ9Vδ2 T_EM_ cells producing IFN-γ and TRAIL.

We also conducted a phase I trial in 10 advanced metastatic breast cancer patients, using the same Zoledronate and IL-2 regimen as in the above study (60), and found that 3 patients who sustained Vγ9Vδ2 T cell numbers achieved either disease stabilization (2 patients) or partial remission (1 patient).

While the above studies by the Wilhelm’s group and our group have used n-BPs and IL-2, Bennouna and colleagues (61) conducted a phase I trial using the synthetic PAg BrHPP with low doses of IL-2 in 28 patients with solid tumors. Patients first received BrHPP alone intravenously (i.v.) and then were treated with BrHPP i.v. in combination with IL-2 s.c. at weekly intervals. The BrHPP and IL-2 treatment was well tolerated and induced *in vivo* dose-dependent Vγ9Vδ2 T cell amplification. Based on these findings and the results from a preclinical study in macaques (62), Bennouna and colleagues conducted a multicentric phase II trial with BrHPP and IL-2 in 45 patients with follicular B-cell lymphoma who had been previously treated with the anti-CD20 mAb Rituximab. The treatment provoked expansion of Vγ9Vδ2 T lymphocytes in 39 out of the 45 patients, which peaked 1 week after the first injection of BrHPP, but declined upon subsequent injections. However, Vγ9Vδ2 T cells acquired the capability to produce IFN-γ and TNF-α and expressed FcγRIII (CD16) which promoted ADCC activity after the second and third injections of BrHPP. Clinical results from 38 patients consisted of 10 instances of complete remission (CR) and 17 overall response rate. Therefore, administration of BrHPP, IL-2 and Rituximab produced very promising results, with limited side effects, overall supporting the potential of combining Vγ9Vδ2 T cell-based therapies with mAbs.

In contrast with these extremely promising results, two other phase I trials have confirmed that the Vγ9Vδ2 T cell-based therapy is well tolerated, but have not shown evidence of antitumor effects. Lang and colleagues (63) have conducted a phase I trial with Zoledronate and IL-2 in 12 patients with metastatic renal cell carcinoma. All patients experienced low grade adverse events, but no clinical response was observed. Rather, the treatment induced a significant decrease of the *in vitro* Vγ9Vδ2 T cell proliferative response in the majority of the patients.

In another study, Kunzmann and coworkers (64) conducted a prospective phase I study with Zoledronate and IL-2 in 21 patients with different advanced malignancies. The regimen was well tolerated and caused a marked *in vivo* activation and IFN-γ production of Vγ9Vδ2 T cells in all evaluable patients, but objective responses (partial remission) were observed only in two patients with acute myeloid leukemia. Interestingly, the lack of clinical response was associated with elevated pretreatment levels of serum vascular endothelial growth factor, which were even increased upon injection of Zoledronate and IL-2.

Finally, a recent prospective, non-randomized Phase I trial, has been conducted in nine young patients with refractory neuroblastoma, which has demonstrated that *in vivo* administration of Zoledronate and IL-2 s.c. can safely expand *in vivo* circulating Vγ9Vδ2 T cells, suggesting that intentional *in vivo* activation of Vγ9Vδ2 T cells might represent a strategy for the treatment of neuroblastoma (65).

## Results from Clinical Trials Based on Adoptive Transfer of *Ex Vivo*-Expanded γδ T Cells

Phase I clinical trials using adoptive transfer of *ex vivo*-expanded γδ T cells have yielded somewhat conflicting results. A survey of these studies in different types of cancer is shown in Table 2.

**Table 2:** Survey of clinical trials based on adoptive transfer of *ex vivo*-expanded γδ cells.

Author	Year	Tumor	Treatment	Reference
Wada et al.	2014	Gastric cancer	Vγ9Vδ2 + Zoledronate	(70)
Abe et al.	2009	MM	Vγ9Vδ2 + Zoledronate + IL-2	(71)
Kobayashi et al.	2007, 2011	RCC	Vγ9Vδ2 + Zoledronate + IL-2	(66, 67)
Nicol et al.	2011	Solid tumors	Vγ9Vδ2 + Zoledronate	(68)
Bennouna et al.	2008	RCC	Vγ9Vδ2 + BrHPP + IL-2	(72)
Wilhelm et al.	2014		Vγ9Vδ2 + Zoledronate + IL-2	(69)
Nakajima et al.	2010	NSCLC	Vγ9Vδ2 + Zoledronate + IL-2	(73)
Sakamoto et al.	2011	NSCLC	Vγ9Vδ2 + Zoledronate + IL-2	(74)

*MM, multiple myeloma; RCC, renal cell cancer; NSCLC, non-small cell lung cancer; BrHPP, bromohydrin pyrophosphate; IL, interleukin*.

Five studies have given results suggesting an antitumor effect of the therapy. Two trials were carried out by Kobayashi’s group in patients with advanced renal cell carcinomas; in one study (66), seven patients received Zoledronate-expanded Vγ9Vδ2 T cells and IL-2 i.v. All patients had mild adverse events, four patients showed a significant *in vivo* expansion and IFN-γ production by Vγ9Vδ2 T cells, but the clinical benefit was moderate, as only three out of seven patients showed delayed tumor doubling time (66). In the second trial from the same group, all 11 patients receiving Zoledronate-expanded Vγ9Vδ2 T cells and IL-2 showed prolonged tumor doubling time (67).

In another trial Nicol and coworkers (68) evaluated the safety and feasibility of the adoptive transfer of Vγ9Vδ2 T cells expanded *ex vivo* with Zoledronate and IL-2, in combination with Zoledronate given i.v. to 18 patients with advanced solid tumors who continued their previously ineffective chemotherapy. No toxicity was reported, and 3 out of the 18 patients had clinical responses (68). Interestingly, authors tracked Vγ9Vδ2 T cells labeled with ^111^In in three patients. The cells localized to the lungs and remained there for 4–7 h after injection and then migrated to the liver and spleen. In one patient with a large metastasis in the left adrenal gland, the cells accumulated in the metastatic site 1 h after injection and remained there until 48 h.

In a fourth trial, four patients with advanced hematological malignancies received haploidentical transplants (69) highly enriched for Vγ9Vδ2 T cells, followed by *in vivo* administration of Zoledronate and IL-2. Three patients showed CR during the 6-month follow-up, while one patient died of an infection 6 weeks after the cell transfusion.

Most recently, Wada and coworkers have conducted a pilot study in seven patients with neoplastic ascites caused by gastric cancer with Vγ9Vδ2 T cells expanded *ex vivo* with Zoledronate and IL-2, administered together with Zoledronate i.v. Weekly Intraperitoneal injection of Vγ9Vδ2 T cells had no severe adverse events and caused a significant reduction of the number of tumor cells in the ascites, which was evident soon after the first cycle of therapy and sustained over time. CT scan also revealed a significant reduction in volume of ascites in two out of the seven patients. Authors conclude that injection of Vγ9Vδ2 T cells can result in the control of malignant ascites in patients for whom no standard therapy is available (70).

In contrast to the above successful studies, several other phase I trials, while showing that Vγ9Vδ2 T cell adoptive therapy is well tolerated, failed to providing evidence of antitumor effects.

Abe et al. (71) conducted a trial in six subjects with MM who received Zoledronate-expanded Vγ9Vδ2 T cells in combination with Zoledronate and IL-2. The treatment was safe but clinical efficacy, as assessed by M-protein serum levels remained at baseline in four patients and increased in two patients, in the absence of between the number of Vγ9Vδ2 T cells injected and clinical outcome.

Bennouna et al. (72) conducted a phase I trial using *ex vivo*-expanded Vγ9Vδ2 T cells in combination with BrHPP and IL-2, in 10 patients with metastatic renal cell carcinoma. Overall, the therapy was well tolerated with only one severe effect, 6 out of 10 patients showed stable disease, but there was no significant antitumor effect.

Finally, in 2 studies of non-small cell lung cancer (NSCLC) involving 10 and 15 patients, respectively (73, 74), who received *ex vivo*-expanded Vγ9Vδ2 T cells and IL-2, there were no objective clinical responses although about one-third to one-half of the patients showed stable disease after therapy. In one study, Nakajima and coworkers (73) treated 10 patients with NSCLC with Vγ9Vδ2 T cells expanded *ex vivo* with Zoledronate and IL-2. The treatment was safe, three patients showed stable disease and five patients showed a progression of the disease 4 weeks after the last treatment. In the other study, Sakamoto and coworkers (74) injected *ex vivo*-expanded γδ T cells in patients with advanced NSCLC. Fifteen patients undergoing treatment with these γδ T cells did not have severe adverse events, all patients remained alive during the study period, but there were no objective responses.

## What Do the γδ T Cell-Based Clinical Trials Teach Us?

Clinical trials exploiting γδ T cells in cancer have been conducted over the past decade, with a good safety profile but variable efficacy. What is clear from these studies is that there is enormous variation in the types of cancer treated, combined with heterogeneity in the protocols used to expand γδ T cells *in vivo* or *ex vivo* for cellular immunotherapy, or in how the immunotherapy was delivered (e.g., PAgs or Zoledronate with or without IL-2, or in combination with other drugs, γδ T cells alone or in combination with activating drugs such as IL-2 and Zoledronate). In addition, several factors may influence the success of γδ T cell-based immunotherapy, which will be discussed in this section.

Immunotherapy strategy based on intentional activation of Vγ9Vδ2 T cells *in vivo* by administration of PAgs or n-BPs and IL-2 has been effective in activating circulating Vγ9Vδ2 T cells, but there is no evidence that this approach reaches tissue-resident γδ T cells or even promotes their recruitment at the tumor site, where they should in fact exert their antitumor activities.

Moreover, patients with several types of tumors have low numbers and unresponsive γδ T cells (75), even if more recent evidences indicate that reductions of γδ T cell numbers and functions might be associated with age and sex and not with the presence of the tumor (76–78).

In addition, a decreased number of circulating Vγ9Vδ2 T cells have been observed as injections of PAgs or Zoledronate and IL-2 progressed, which was accompanied by a lower response of peripheral blood Vγ9Vδ2 T cells to PAgs.

The precise mechanism underlying this phenomenon remains unknown and further investigations are thus necessary. Among the possibilities, activation-induced Vγ9Vδ2 T cell anergy has been frequently reported (75), possibly due to inadequate signals delivered during activation, exposure to suboptimal PAgs concentration or from Vγ9Vδ2 T cell intrinsic features.

A recent clinical trial of Zoledronate given i.v. to cancer-free patients showed that the inflammatory-type side effect of Zoledronate (flu-like syndrome) could be easily predicted by analyzing *in vitro* production of IFN-γ by Zoledronate-stimulated peripheral blood mononuclear cells (79). In agreement with these data, we and others have shown that repeated i.v. injections of Zoledronate was accompanied by decrease of circulating Vγ9Vδ2 T_CM_ cells and reduction of their proliferative responses *in vitro* (79, 80). Circulating neutrophils may also contribute as they take up Zoledronate and produce hydrogen peroxide that inhibits T cell proliferation (81). Finally, repeated stimulation of Vγ9Vδ2 T cells may also cause terminal differentiation and exhaustion (82–84).

Immunoevasion strategies can be exploited by cancer cells to escape recognition and attack by Vγ9Vδ2 T cells. Indeed, several evidences demonstrate that cancer cells acquire the capability to inhibit immunological checkpoints using several different strategies. However, a very recent study has shown that Vγ9Vδ2 T cells express very low programmed death-1 (PD-1) compared with conventional αβ CD8 and CD4 T cells, which was markedly up-regulated over the first 4 days of exposure to Zoledronate and IL-2 *in vitro* but by day 7 dropped nearly to baseline (85). While these results suggest that Vγ9Vδ2 T cells may circumvent the PD-1/PD-1L checkpoint *in vivo*, Hayday and coworkers found that Vγ9Vδ2 T cells express another negative checkpoint receptor, TIGIT, upon *in vitro* activation, thus providing an additional opportunity to cancer cells to escape Vγ9Vδ2 T cell-mediated elimination (Hayday, unpublished results). Evasion strategies that specifically impair Vγ9Vδ2 T cell functions can involve diverse immunosuppressive mediators produced in the tumor microenvironment, as, for example, transforming growth factor-β, prostaglandins, kynurenins, or potassium (86–89).

All of the above pitfalls may be partly overcome by utilization of the adoptive cell transfer of *ex vivo*-expanded Vγ9Vδ2 T cells, which thus seems to be a more effective procedure. However, the problem appears to be how to sustain the levels and functions of the transferred Vγ9Vδ2 T cells. In metastatic renal cell carcinoma, two groups reported superior efficacy when Vγ9Vδ2 T cells were administered with Zoledronate and/or IL-2, as compared to Vγ9Vδ2 T cells administered alone (67, 90).

While the aforementioned trials utilized patients’ autologous peripheral blood-derived Vγ9Vδ2 T cells, a recent study by Wilhelm and colleagues (69) utilized Vγ9Vδ2 T cells from haploidentical donors; this treatment did not cause graft-versus-host disease and was clinically effective as three out of four patients achieved CR (69). Vγ9Vδ2 T cells from the haploidentical donor persisted for 28 days and expanded *in vivo* following injection of Zoledronate and IL-2.

Other studies have shown that it is possible to sustain injected Vγ9Vδ2 T cells without IL-2 supplementation, probably relying on IL-15 (91) or on IL-18 (92, 93) spontaneously produced by the host.

## Can We Improve γδ T Cell-Based Tumor Immunotherapy?

**Figure 2:**
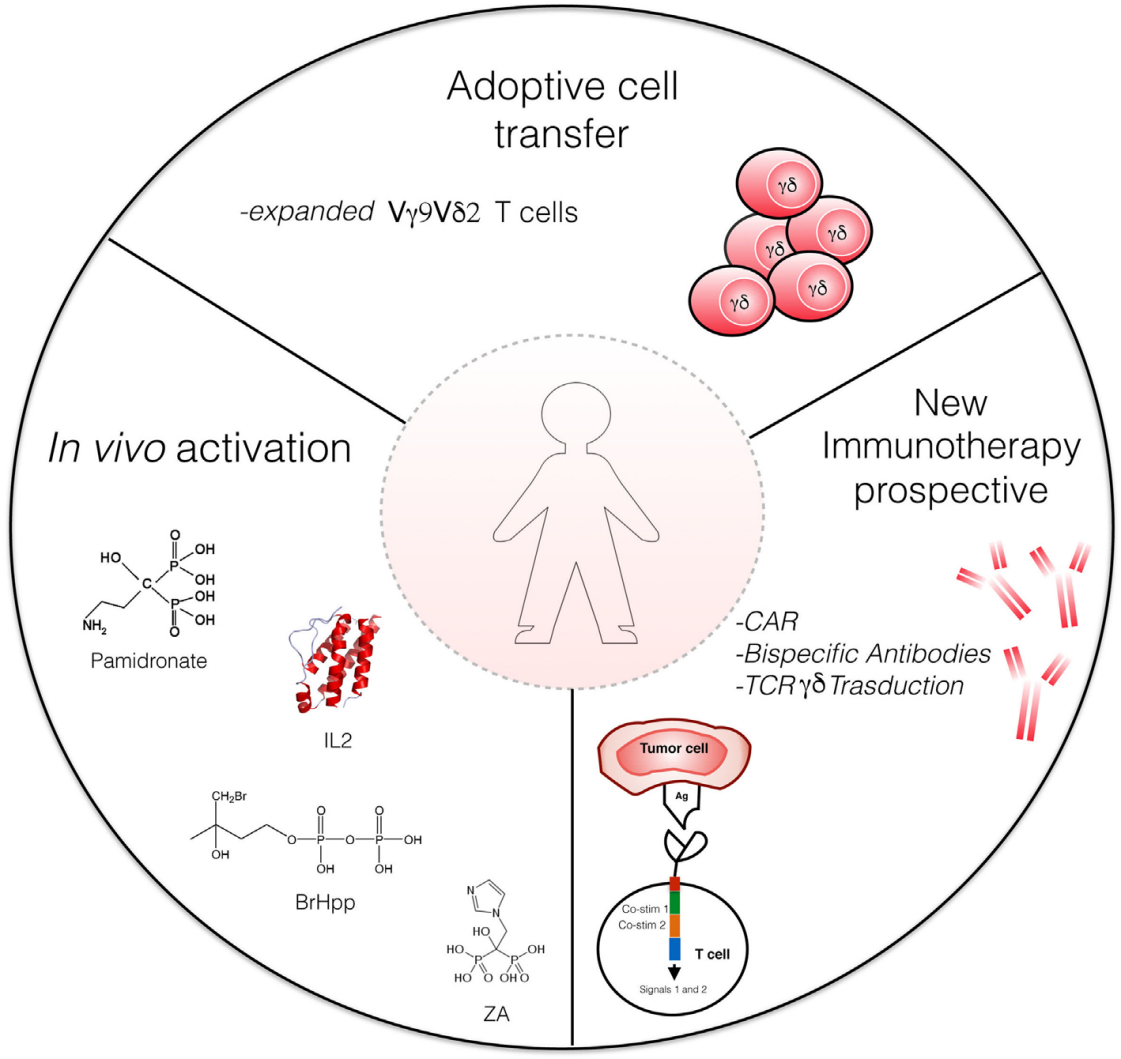
Strategies for γδ T cell-based immunotherapy. Actual strategies include adaptive cell transfer of γδ T cells expanded *in vitro* with Zoledronate and interleukin (IL)-2, and *in vivo* activation of Vγ9Vδ2 T cells by phosphoantigens [e.g., bromohydrin pyrophosphate (BrHPP)] or aminobisphosphonates (Zoledronate) and low-dose IL-2. Novel γδ T cell-based therapeutic strategies involve bispecific antibodies and CAR-T cells. ZA, Zoledronate acid; CAR, chimeric antigen receptors.

γδ T cells can be redirected to the cancer cell using antibodies (Figure 2). This can be achieved, for instance, by using bispecific antibodies, in which one binding site recognizes a tumor-specific cell surface molecule (for example, EpCAM or HER2/neu) and the other binding site targets CD3 or the Vγ9 chain of the Vγ9Vδ2 TCR; such bispecific antibodies have been demonstrated effective in preclinical models (94, 95).

As a variant of the bispecific antibody technology, Zheng et al. (96) prepared a chimeric molecule in which the variable portion derived from the extracellular domains of a Vγ9Vδ2 TCR (cloned from a Vγ9Vδ2 T cell infiltrating ovarian cancer) and the constant region was the fragment crystallizable (Fc) domain of human IgG1. This chimeric construct bound to several ovarian cancer cells, recognizing a yet unknown antigen and promoted the killing of the cells *via* ADCC mediated by binding of the Fc region of the chimeric construct to CD16.

Wesch and colleagues (97) developed recombinant immunoligands consisting of a CD20 single-chain variable fragment (scFv) linked to MICA or ULBP2 and found that both constructs promoted the cytotoxic activity of *ex vivo*-expanded γδ T cells (containing both Vδ1 and Vδ2 T cells) against CD20-positive lymphoma cells. Importantly, these two immunoligands mediated the killing of chronic lymphocytic leukemia cells isolated from patients by γδ T cells, which was even enhanced by the PAg BrHPP. Thus, the utilization of recombinant immunoligands which engage NKG2D, with or without simultaneous TCR triggering, may represent an attractive strategy to enhance antitumor cytotoxicity of γδ T cells.

Another approach consists in lentiviral-mediated transduction of T cells with chimeric antigen receptors (CARs; Figure 2). CARs are usually derived from scFvs of antibodies specific for tumor antigens, thus enabling the CAR-transduced T cells to recognize tumor epitopes independently on their TCR [reviewed in Ref. (98)].

To date, most CAR utilize αβ T cells, but γδ T cells are also an appealing target, due to their antitumor effector functions.

Deniger et al. (99) have transduced polyclonal γδ T cells with a CD19-specific CAR which conferred the capability to efficiently kill CD19^+^ leukemia cells. The CAR technology has been combined with the generation of induced pluripotent stem cells from human peripheral blood T cells (100). Such cells showed a very similar phenotype to γδ T cells and exerted antitumor activity.

T cells can be redirected to tumors by lentiviral-mediated transduction with an exogenous TCR of known anticancer specificity, following adoptive transfer into patients. Typically, the vast majority of studies have involved transduction of an αβ TCR of well known antitumor specificity into another αβ T cell (101). The major problem with this strategy is the risk of mispairing between the endogenous and exogenous TCR α and β chains, resulting in receptors with autoreactive specificities (102, 103).

γδ T cells offer an attractive solution to this problem, in the sense that a given tumor-specific αβ TCR can be introduced into γδ T cells without the risk of mispairing (104, 105). Another advantage is that γδ T cells transduced with an αβ TCR retain the functionality of their original TCR, thereby responding rapidly upon antigen stimulation (106).

The main obstacle associated with the αβ TCR transfer, is that γδ T cells do not express CD4 or CD8 co-receptors, which are required for efficient recognition of peptide–MHC complexes on target cells. This implies that co-transduction with a co-receptor (107) or use of very high affinity TCRs (108) would be desirable to enhance antitumor activity of αβ-transduced γδ T cells. It is also possible to transduce peripheral lymphocytes (both γδ and αβ) with a specific γδ TCR, as successfully demonstrated by Zhao and coworkers (109, 110).

Finally all γδ T cell-based clinical trials in patients with hematologic and solid tumors have relied on the utilization of Vγ9Vδ2 T cells. Vδ1 T cells are typically less susceptible to activation-induced exhaustion and in theory could persist long after adoptive transfer, providing the host with a durable antitumor immune response (111). Moreover, as Vδ1 T cells express several NK receptors and possess a highly cytotoxic potential (8), they may constitute a potent therapeutic lymphocyte population that could be exploited in alternative to, or in addition to Vγ9Vδ2 T cells. Accordingly, Silva Santos and coworkers (112) have recently developed a clinical-grade method to selectively expand Vδ1 T cells. The expanded Vδ1 T cells efficiently inhibited tumor growth and prevented dissemination in xenograft models of leukemia, thus providing a preclinical proof of principle for application of Vδ1 T cells in adoptive immunotherapy of cancer.

## Conclusion

Overall, studies performed to date have clearly demonstrated that γδ T cell-based tumor immunotherapy is safe, but clinical performance has been inconsistent (31). Identification of the ligands recognized by Vδ1^+^ and Vδ2^+^ T cells, the antigen and cytokine requirements for their differentiation and survival, and the interactions they establish with tumor cells and other different components of the tumor microenvironment, will lead to a better understanding of how γδ T cells work and to properly harness these cells for effective and durable tumor immunotherapy.

## Author Contributions

EG, GC, and GG provided clinical samples and patient’s data. EP and GP analyzed data in the literature and prepared figures. FD and SM wrote the manuscript.

## Conflict of Interest Statement

The authors declare that the research was conducted in the absence of any commercial or financial relationships that could be construed as a potential conflict of interest. The reviewer GA and handling editor declared their shared affiliation.

## Abbreviations

ADCC, antibody-dependent cell-mediated cytotoxicity; BTN, butyrophilin; BrHPP, bromohydrin pyrophosphate; CAR, chimeric antigen receptor; CSC, cancer stem cell; DR5, death receptor 5; Fc, fragment crystallizable; FcR, fragment crystallizable receptor; Fv, variable fragment; GMP, good manufacturing practice; HMGCR, 3-hydroxy-3-methylglutaryl-CoA reductase; IFN, interferon; IL, interleukin-; iPSC, inducible pluripotent stem cells; mAbs, monoclonal antibodies; MHC, major histocompatibility complex; MICA, MHC class I-related molecule A; MICB, MHC class I-related molecule B; MM, multiple myeloma; n-BP, aminobisphosphonate; NCR, natural cytotoxicity receptors; NHL, non-Hodgkin lymphoma; NKG2D, natural-killer group 2, member D; NSCLC, non-small cell lung cancer; PAg, phosphoantigen; PD-1, programmed death-1; PD-L1, programmed death-ligand 1; TCR, T cell receptor; TFH, follicular T helper; TGF, transforming growth factor; Th, T helper; TIGIT, T-cell immunoreceptor with Ig and ITIM domains; TNF, tumor necrosis factor; TRAIL, TNF-related apoptosis-inducing ligand; Treg, T regulatory; ULBP, UL16-binding protein; VEGF, vascular endothelial growth factor.
